# Reduction of cytotoxicity of benzalkonium chloride and octenidine by Brilliant Blue G

**DOI:** 10.17179/excli2014-556

**Published:** 2015-01-21

**Authors:** Melinda Bartok, Rashmi Tandon, Gabriela Alfaro-Espinoza, Matthias S. Ullrich, Detlef Gabel

**Affiliations:** 1 Molecular Life Science Research Center, Jacobs University Bremen, Campus Ring 1, 28759, Bremen, Germany

**Keywords:** Brilliant Blue G, Brilliant Blue R, Benzalkonium chloride, Octenidine dihydrochloride, P2x7 receptor antagonist

## Abstract

The irritative effects of preservatives found in ophthalmologic solution, or of antiseptics used for skin disinfection is a consistent problem for the patients. The reduction of the toxic effects of these compounds is desired. Brilliant Blue G (BBG) has shown to meet the expected effect in presence of benzalkonium chloride (BAK), a well known preservative in ophthalmic solutions, and octenidine dihydrochloride (Oct), used as antiseptic in skin and wound disinfection. BBG shows a significant protective effect on human corneal epithelial (HCE) cells against BAK and Oct toxicity, increasing the cell survival up to 51 % at the highest BAK or Oct concentration tested, which is 0.01 %, both at 30 min incubation. Although BBG is described as a P2x7 receptor antagonist, other selective P2x7 receptor antagonists, OxATP (adenosine 5’-triphosphate-2’,3’-dialdehyde) and DPPH (N’-(3,5-dichloropyridin-4-yl)-3-phenylpropanehydrazide), did not reduce the cytotoxicity of neither BAK nor Oct. Therefore we assume that the protective effect of BBG is not due to its action on the P2x7 receptor. Brilliant Blue R (BBR), a dye similar to BBG, was also tested for protective effect on BAK and Oct toxicity. In presence of BAK no significant protective effect was observed. Instead, with Oct a comparable protective effect was seen with that of BBG. To assure that the bacteriostatic effect is not affected by the combinations of BAK/BBG, Oct/BBG and Oct/BBR, bacterial growth inhibition was analyzed on different Gram-negative and Gram-positive bacteria. All combinations of BAK or Oct with BBG hinder growth of Gram-positive bacteria. The combinations of 0.001 % Oct and BBR above 0.025 % do not hinder the growth of *B. subtilis*. For Gram-negative bacteria, BBG and BBR reduce, but do not abolish, the antimicrobial effect of BAK nor of Oct. In conclusion, the addition of BBG at bacterial inhibitory concentrations is suggested in the ready-to-use ophthalmic preparations and antiseptic solutions.

## Introduction

Brilliant Blue G (BBG) is widely used as a dye for proteins in gel electrophoresis. Its power to stain proteins has led to its application in vitreoretinal surgery for the staining of the internal limiting membrane (ILM), as it showed no toxic effects at the clinically suggested concentration, 0.025 %, while showing good staining ability (Enaida et al., 2006[6]). Later, BBG was found to be a great asset also in the treatment of the spinal cord injury, because it reduced the local inflammatory responses, showed protective effects towards the spinal cord neurons and improved the motor recovery (Marcillo et al., 2012[18]; Peng et al., 2009[22]). The authors paired the physiological function of BBG with its activity as a P2x7 receptor antagonist as the spinal cord neurons express this receptor abundantly at their surface. 

In previous studies, we showed that BBG protects ARPE retinal pigment epithelium cells against the toxicity of trypan blue (TB), which is another widely used dye for the staining of the ILM in eye surgery (Awad et al., 2013[2]). This observation was unexpected, and no clear mechanism was proposed. Also, it was not known whether the protective effect of BBG was limited to TB, or whether it was more universal. Brilliant Blue R (BBR) is a dye very similar to BBG, which differs only by the absence of two methyl groups. BBR, in contrast to BBG, has never been used medically. In the literature, BBR is used only as a sensitive protein stain in polyacrylamide gel electrophoresis (Servaites et al., 2012[25]). In this study we have tested the cytotoxicity of benzalkonium chloride (BAK) and octenidine (Oct) in combination with BBG or BBR in order to see whether there is any significant reduction in the toxicity of the compounds on human corneal epithelial cells (HCE). BAK is a widely used preservative in ophthalmic drops, even though it is known to have cytotoxic effects and causes easily inflammation in the eye surface (Ammar and Kahook, 2011[1]; Dutot et al., 2006[5]; Liang et al., 2012[16]; Paimela et al., 2012[21]). Oct is an antiseptic agent for skin, mucous membranes and wounds, and is used in many preparations as a replacement for other antiseptics, because it shows a significantly higher efficiency already at very low concentrations (Hübner et al., 2010[9]; Koburger et al., 2010[13]). At the clinically and industrially used concentrations, both compounds show high cytotoxicity against mammalian cells. After only 5 minutes incubation with HCE cells, less than 20 % of the cells were found to be metabolically active. Interestingly, we found a high protective effect of BBG against the cytotoxic action of BAK and Oct, without an excessive reduction of the bacteriostatic effect. 

## Materials and Methods

### Materials

Dulbecco’s Modified Eagles Medium with Ham’s F12 (DMEM + F12), BAK, BBG, BBR, OxATP (adenosine 5’-triphosphate-2’,3’-dialdehyde) were purchased from Sigma-Aldrich (Schnelldorf, Germany). Oct was provided by Schülke & Mayr GmbH (Norderstedt, Germany). HCE cells were from Riken Bioresource Center (Tsukuba, Japan). WST-1 cell proliferation reagent was from Roche Diagnostics (Mannheim, Germany). Fetal bovine serum (FBS) and penicillin/streptomycin (Pen/Strep) were from Biochrom (Germany). Human epithelial growth factor (hEGF), insulin, amphotericin B, and L-glutamine were from Sigma-Aldrich (Steinheim, Germany), TrypLE Express was from Gibco (USA). DPPH (N’-(3,5-dichloropyridin-4-yl)-3-phenylpropanehydrazide) was synthesized as described by Lee et al. (2012[15]). The medium MHB (Mueller-Hinton Bouillon) was from Carl Roth (Germany).

### Test solutions

BBG was used between 0.0025 and 0.075 %, in combination with either BAK between 0.001 and 0.01 %, or Oct between 0.002 and 0.01 %. BBR was tested between 0.0025 and 0.05 % in the presence of BAK at 0.004 % and Oct at 0.003 %. As negative control PBS was used. Each of these concentrations were tested also alone on HCE cells as well as on the below mentioned bacterial strains.

### Cell culture and cell viability assay

HCE cells were cultivated in DMEM + F12 media, supplemented with 15 % FBS, 1 % Pen/Strep, 25 µg/ml amphotericin B, 5 µg/ml insulin, 10 ng/ml hEGF and 2 mM L-glutamine, at 37°C and 5 % CO_2_. The cells were passaged by trypsinization with TrypLE Express and seeded at a density of 20,000 cells/well in a 96 well, flat bottom plate and grown for 48 h prior to experiment. After the cells reached confluence, they were incubated with 50 µl/well of different test solutions for 5, 30 and 60 min at 37 °C and then washed 3 times with PBS (w/o Ca^2+^ and Mg^2+^). One hundred µl of diluted WST-1 cell proliferation reagent (diluted 1:4 in PBS, then 1:10 in cell culture medium) was added to each well and incubated with the cells at 37 °C for 4 h. The WST-1 reagent, which is a tetrazolium salt, is reduced to a red formazan dye by the mitochondrial dehydrogenases of metabolically active cells. We had checked previously that the readings for this dye are not influenced by any remaining BBG (Awad et al., 2013[2]). The amount of formazan dye formed was taken as a measure of cell survival. The absorbance of the plate was measured in an MR5000 plate reader (Dynatech) at 450 nm. All test solutions were tested in three independent experiments, with 6 to 12 measurements for each experiment.

### Determination of inhibitory concentrations for bacterial cells

The Gram-positive bacteria *Bacillus subtilis, Clavibacter michiganensis* and* Paenibacillus sp.* as well as the Gram-negative bacteria *Escherichia coli* DH5α, *Pseudomonas* *putida* DSM 291 and *Vibrio sp.* Gal12 were used in the assay. The antimicrobial activities of the compound mixtures were assayed in micro-titer plates. For this, 100 µl of MHB were aliquoted into each well of the micro-titer plate, followed by 20 µl of a mixture of antimicrobial compounds and 100 µl of the bacterial suspension (approximately 2·10^6^ cells/ml). Deionized water was used as negative control. All micro-titer plates were incubated overnight at 28 °C, except for* E. coli* plates, which were incubated at 37 °C. Following overnight incubation, the plates were examined for visible bacterial growth evidenced by the turbidity. Where no turbidity was seen, we assumed that there is no bacterial growth. For each compound, the assay was performed in triplicate in three independent experiments and in accordance with Jorgensen and Turnidge (2007[12]). 

## Results

**Figure 1:**
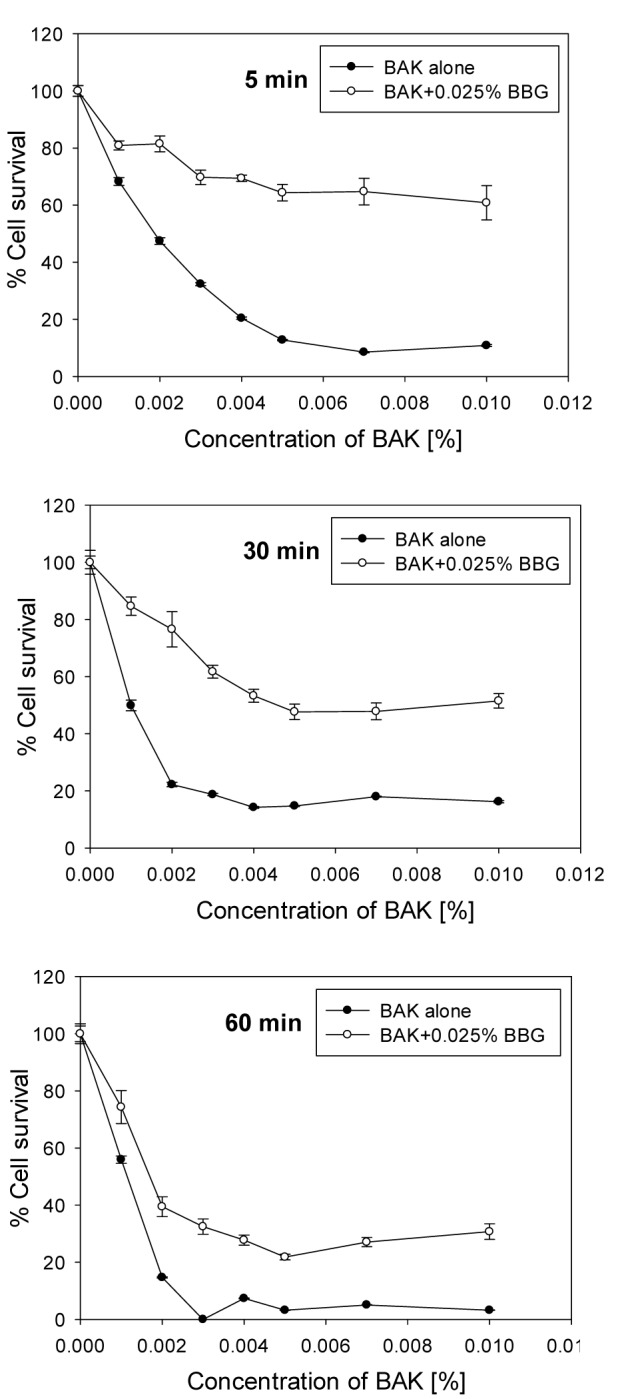
Survival of HCE cells after exposure for 5, 30 and 60 minutes to different concentrations of BAK in PBS (“BAK alone”) and in combination with 0.025 % BBG. The error bars represent the standard deviations of 6 to 12 replicates performed within the same experiment.

To investigate the influence of the time and the concentrations of BBG, BAK and Oct on the HCE cell viability, as well as their impact on the bacterial growth inhibition, the cells were exposed to different concentrations of these compounds. Our results showed that BAK was highly toxic for HCE cells. Already in concentrations of 0.001 % and 5 min incubation, 30 % cell loss was observed. For longer exposure, as would be used in wound treatment, concentrations of 0.002 % reduced the cell survival to 20 % or less. The inclusion of BBG, in the concentration of 0.025 % used clinically for staining procedures, reduced the toxicity of BAK considerably (Figure 1(Fig. 1)). This protective effect of BBG led to an increase of cell survival, with 50 % at 5 min, with 35 % at 30 min and with 27 % at 60 min incubation at a concentration of BAK 0.01 %, the highest concentration tested in this study. 

**Figure 2:**
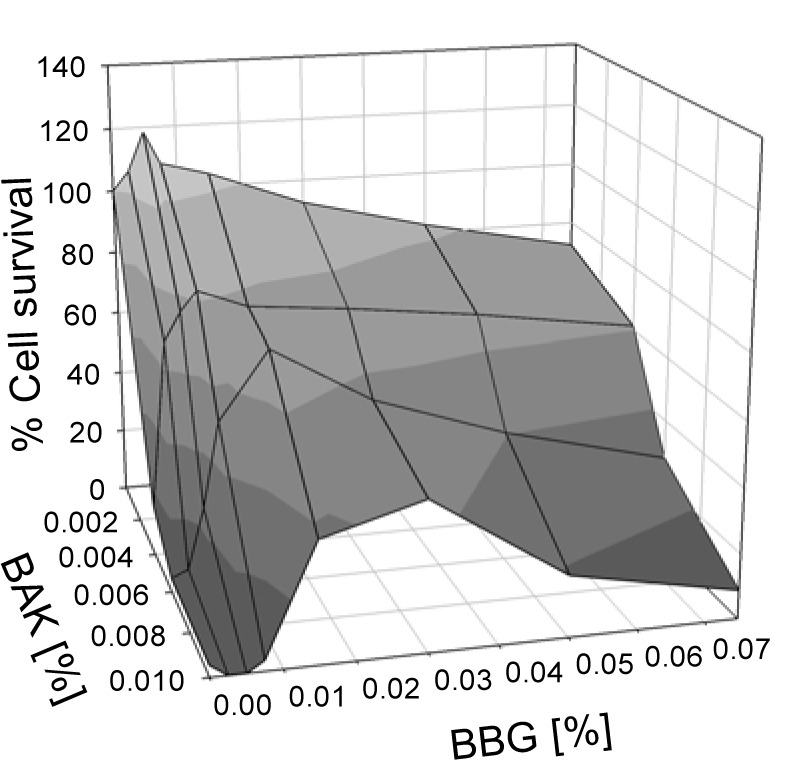
Cell survival of HCE cells after exposure for 30 min to combinations of BAK and BBG in different concentrations.

The cell survival after exposure to BAK in the presence of different BBG concentrations is shown in Figure 2(Fig. 2). Even at low BBG concentrations, the compound showed significant increase of the cell survival: at 0.007 % BBG, 67 % of the cells survived exposure to 0.004 % BAK, and 48 % survived when exposed to 0.006 % BAK. Without BBG, cell survival at these concentrations of BAK was around 15 % (Figure 1(Fig. 1)). At higher BAK concentrations, higher BBG concentrations have to be chosen; for example at 0.01 % BAK with 0.015 % BBG the cell survival increased to 37 % and with 0.030 % BBG to 46 %, at 30 min incubation, whereas in the absence of BBG, cell survival of only a few percent was seen.

**Figure 3:**
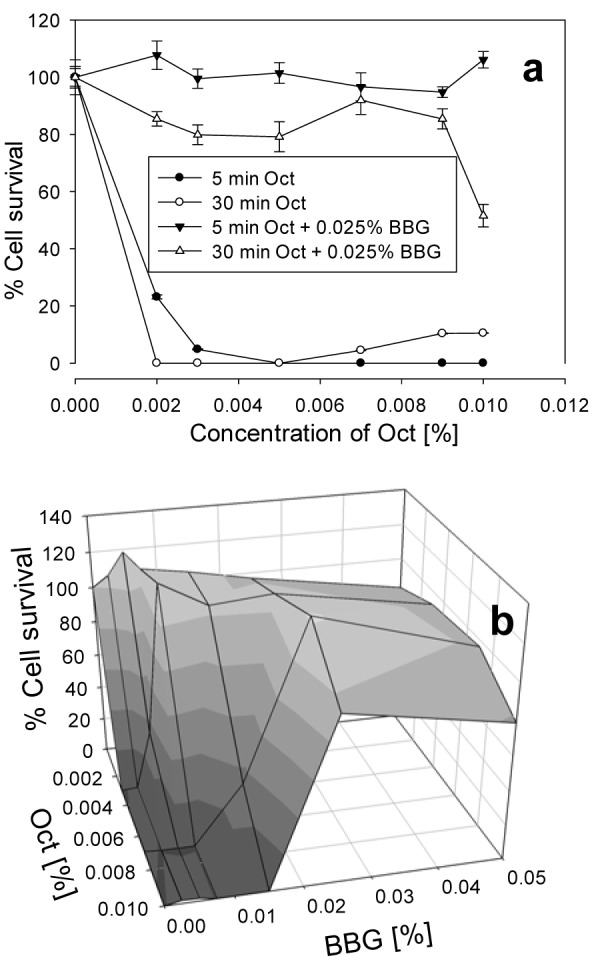
Cell survival of HCE cells after exposure for 5 and 30 min to different concentrations of Oct with and without 0.025 % BBG (a) and after exposure for 30 min to different combinations of BBG and Oct (b). The error bars represent the standard deviations of 6 to 12 replicates performed within the same experiment.

Oct is an antiseptic agent which, in comparison with BAK, had a higher bacteriostatic effect, but at the same time was more toxic to the cells, as shown in Figure 3a(Fig. 3). BBG protected the cells against Oct toxicity even more than against BAK toxicity. After 5 min incubation with 0.009 % Oct and 0.025 % BBG, the cell survival increased to 95 %, and after 30 min incubation, to 85 %, while in the absence of BBG, Oct led to almost complete cell death. In order to find the best combination between Oct and BBG, different concentrations of BBG and Oct were tested in mixture for 30 min incubation (Figure 3b(Fig. 3)). For 0.003 % Oct, already 0.007 % BBG abolished the toxicity of the antiseptic. For 0.007 % Oct, a higher concentration of BBG, namely 0.025 %, had to be taken, in order to achieve the same effect. At 0.01 % Oct concentration, the minimal concentration of BBG needed is 0.025 %, and the cell survival increased to 95 %. 

**Figure 4:**
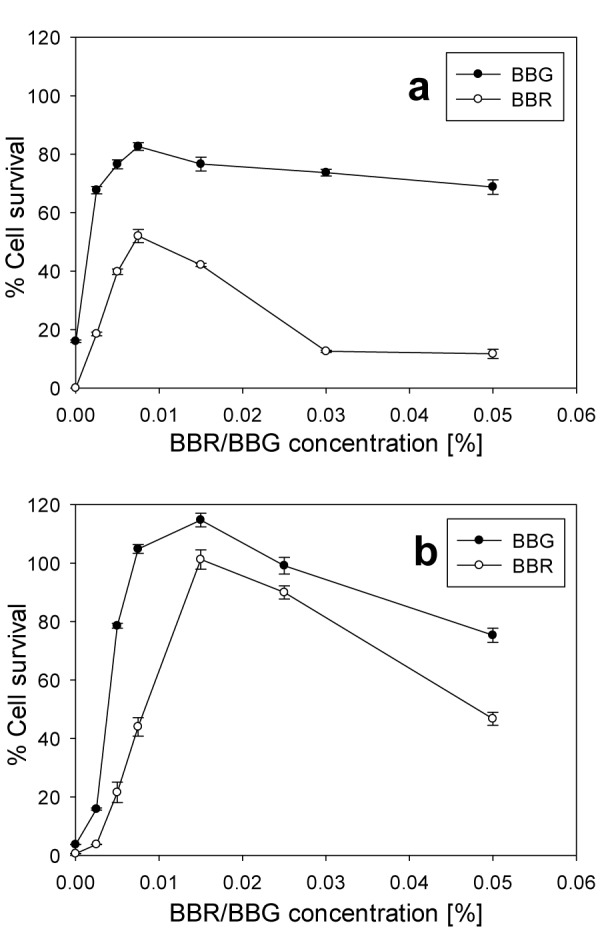
Cell survival of HCE cells after exposure for 30 min to BBR or BBG, in the presence of 0.004 % BAK (a) or 0.003 % Oct (b). The error bars represent the standard deviations of 6 to 12 replicates performed within the same experiment.

BBR is a dye which differs from BBG only by the absence of two methyl groups. This difference changed significantly the protective activity of BBR against BAK and only slightly against Oct. At 0.004 % of BAK there was no considerable cell survival in presence of BBR at 0.025 % or higher, while with BBG, the cell survival increased up to 74 % (Figure 4a(Fig. 4)). The highest protective effect of BBR was obtained at 0.0075 %, where the HCE cell survival increased to 52 %. This was considerably less than the protective effect of BBG at the same concentration, which was 83 %. At 0.003 % of Oct BBR showed comparable protective effect with that of BBG (Figure 4b(Fig. 4)). The highest cell survivals at this Oct concentration was found at 0.015 % and 0.025 % BBR concentrations, which was 102 % and 90 %, respectively. While with BBG at the same concentrations the cell survival was 115 % and 100 %, respectively. Above 0.05 % of BBR or BBG, in presence of Oct, the HCE cells showed a considerable decrease in cell survival.

To determine if the protective effect of BBG on antiseptics was due to its P2x7 receptor antagonist activity, other reported selective P2x7 receptor antagonist were tested in this study. One of them was OxATP. It is known as an irreversible P2x7 receptor antagonist, which blocks human P2x7 receptors at 10 µM concentration (Hibell et al., 2001[8]; Wang et al., 2004[29]). The other antagonist was DPPH, recently synthesized by Lee et al. (2012[15]), which has an IC_50_ of 0.65 µM with ethidium bromide uptake assay. Neither OxATP, used between 2 µM and 2mM (preincubated for 30 min with HCE cells prior to exposure to BAK), nor DPPH, used between 0.1 µM and 10 µM, showed any protective effect against BAK toxicity, when BAK is present between 0.001 and 0.01 %. Oct was tested between 0.003 % and 0.01 % only in presence of DPPH and no protective effect of the P2x7 receptor antagonist was found (data not shown).

### Bacteriostatic effect of Oct and BAK in the presence of BBG and BBR

**Figure 5:**
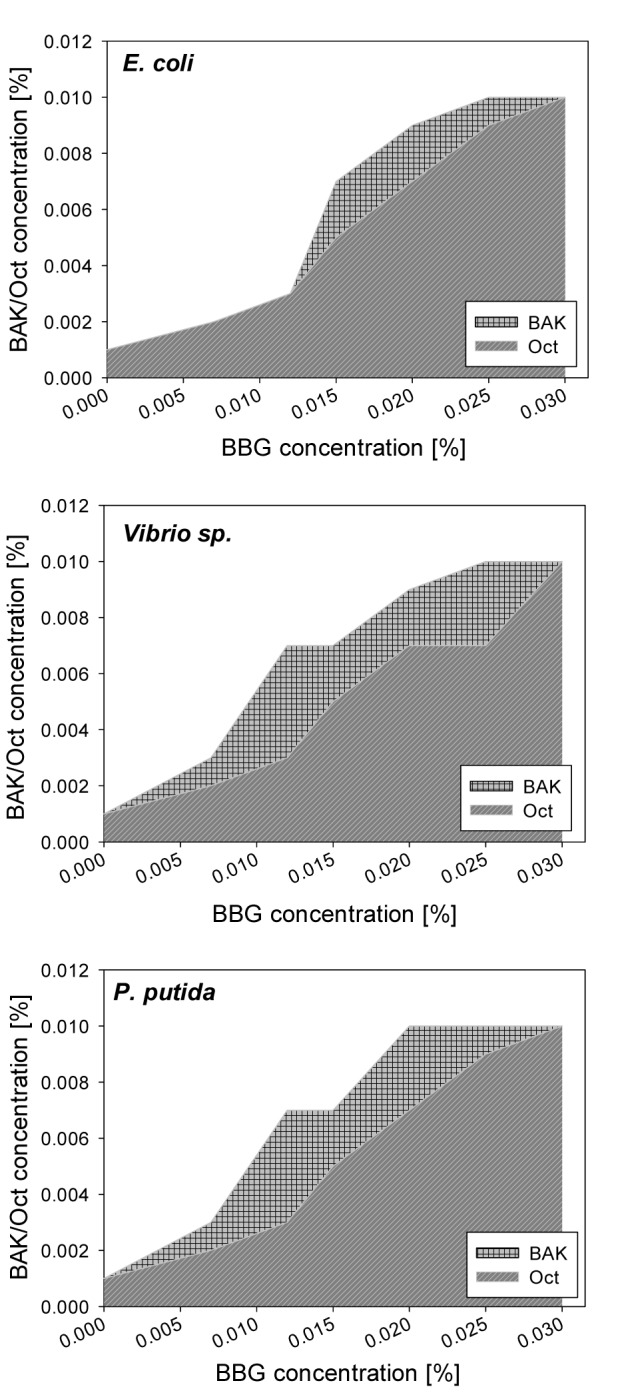
Concentrations of BAK and Oct required to inhibit Gram-negative bacterial growth, when BBG is present. At combinations within the shaded area bacterial growth is observed; combinations above the shaded areas prevent bacterial growth.

**Figure 6:**
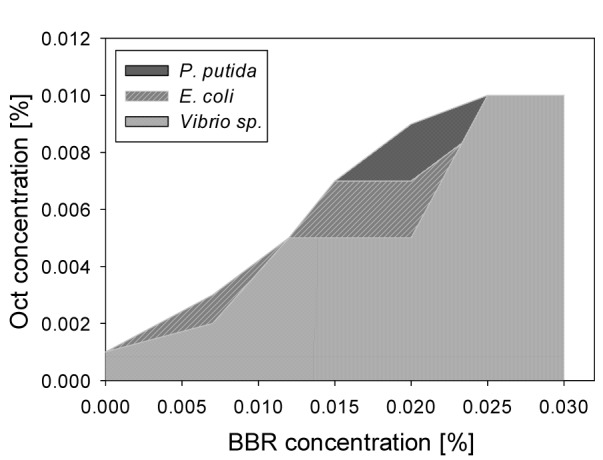
Concentrations of BAK and Oct required to inhibit Gram-negative bacterial growth, when BBR is present. At combinations within the shaded area bacterial growth is observed; combinations above the shaded areas prevent bacterial growth.

Mixtures of BAK or Oct with BBG and BAK or Oct with BBR were used to determine the susceptibility of diverse bacterial strains towards the compound combinations. The BAK and Oct at concentrations between 0.002 % and 0.01 % inhibited the growth of all tested Gram-positive bacterial strains, even when mixed with the highest applied BBG concentration. In general, Gram-positive bacteria were more susceptible towards the compounds than Gram-negative bacteria. The inhibitory concentrations for Gram-negative bacterial growth were summarized in Figure 5(Fig. 5) and Figure 6(Fig. 6). All tested Gram-negative bacteria showed similar responses towards Oct and BAK when the same concentration of BBG was used. These results suggested that the growth of different bacterial organisms were efficiently inhibited by the use of 0.015 % BBG with 0.009 % BAK or 0.007 % Oct, as well as by the use of 0.007 % BBG with 0.005 % BAK or 0.003 % Oct. 

BBR was assayed in combination with Oct on the Gram-negative and Gram-positive bacterial strains used before, to test whether BBR has the same bacterial inhibitory effect as BBG. The results showed that the bacterial growth of the Gram-positive bacterium, *B. subtilis* was not inhibited in presence of high BBR concentrations, i.e. 0.025 % or 0.030 % combined with 0.001 % Oct there. For the Gram-negative bacteria, increasing Oct concentrations were required with increasing BBR concentrations, similar to BBG (see Figure 6(Fig. 6)). The results showed that the growth of all tested bacterial strains were inhibited by the use of 0.007 % BBR with 0.005 % of Oct, 0.012 % BBR with 0.007 % Oct and 0.020 % BBR with 0.009 % Oct. Combinations between BBR and BAK have not been investigated, because no significant protective activity of BBR on human cell lines was seen in presence of BAK.

The concentrations of BAK used in this study are in the range of concentrations used in the commercially available eye drops, 0.004-0.02 % (Liang et al., 2012[16]), where 0.025 % BBG exerted a significant cell viability increase on HCE cells. To achieve bacterial growth inhibition, as well as a significant increase of the cell viability, higher concentrations of BBG required higher BAK concentrations. The best combinations between these two compounds were 0.015 %

BBG for BAK between 0.008 % and 0.01 %, and 0.007 % BBG for BAK between 0.004 % and 0.006 %. The HCE cell viability increased between 40-60 % in the first set of combinations between BBG and BAK, and with 50-80 % in the second case.

Oct was used in this study between 0.002 % and 0.01 %, because according to Hübner et al., (2010[9]) 22.5 mg/l of OPE (Oct with phenoxyethanol) was sufficient to reduce with 3log10 the bacterial growth after 30 min incubation. At these concentrations of Oct, 0.025 % BBG increased the HCE cell survival with up to 100 % at 30 min incubation. Also BBR at the same concentration and incubation time increased the HCE cell survival up to 90 %, when 0.003 % Oct was present. To reduce the toxicity of the antiseptic agent, without affecting the bacterial inhibitory effect, we suggest the following combinations: 0.025 % of BBG with 0.01 % Oct; 0.015 % BBG with 0.007 % Oct; and 0.007 % BBG with 0.003 % Oct. With these combinations, the HCE cell survival increased with 35-115 %.

## Discussion

The inflammatory responses of the eyes or skin due to the repeated contact with preservatives and antiseptics used in different eye drops, wound disinfectants or cosmetical products, are unwanted side effects, and are present in many patients using these products on a daily basis. The reason for these inflammatory effects is the high toxicity of the compounds on the target cells. Therefore, reducing the toxicity of antiseptics and preservatives on human tissues, while maintaining their bacteriostatic effect, is desired. Ways to reduce these inflammatory effects, by combination of bacteriostatic agents with protective agents, were not yet investigated.

We found that the protective effect of BBG against BAK and Oct toxicity on human eye cells is remarkable. However, there are only limited numbers of possible combinations between different concentrations of BBG and BAK or Oct, at which the bacterial growth is inhibited. Very high concentrations of BBG, 0.03 % or higher, in presence of either of the two antiseptics, at the tested concentrations, does no longer inhibit the bacterial growth of Gram-negative bacteria. BBR showed protective effect only against Oct toxicity, however this effect is little lower than the one caused by BBG.

The microbiology experiments showed that the mixtures between BAK with BBG, Oct with BBG and Oct with BBR had stronger inhibitory effects against Gram-positive than against Gram-negative bacterial cells. Since the cell wall composition in both groups of bacteria differs remarkably (Silhavy et al., 2010[26]), the hydrophobic outer membrane of Gram-negative bacteria potentially might prevent the compounds to efficiently enter the cells. However, hydrophilic features of the compounds might aid the entrance of the antimicrobials into Gram-positive cells thus making them more susceptible. Previously McDonnell and Russell (1999[19]) and Fazlara and Ekhtelat (2012[7]) reported that BAK is more effective on Gram-positive bacterial organisms. They also mentioned that the mechanism of action of BAK, as a cationic quaternary ammonium compound, is based on the interaction of the negatively charged bacterial surface with the positively charged headgroup of BAK. After the binding on the bacterial surface, BAK will enter the cell wall and cause its disruption and leakage of the cytoplasmic material to the outside. The mechanism of action of Oct has not been described yet, but we assume that it is similar to BAK. Both of them have an amphiphilic structure, but Oct is a more complex molecule, with two cationic centers and long hydrophobic chains at both ends of the molecule. Oct and BAK had previously been successfully tested as antimicrobial compounds (Hübner et al., 2010[9]; Sedlock and Bailey, 1985[24]). In comparison with BAK, Oct showed a higher efficiency against Gram-negative bacteria. 

We have demonstrated here that BBG reduces the antibacterial effect of Oct and BAK for Gram-negative bacteria. Nevertheless, the use of certain concentration combinations of the compounds, presented in Figure 5(Fig. 5), will efficiently inhibit the growth of bacteria in eye drops, skin, mucous membranes and wounds disinfectants, while protecting the epithelial cells against the cytotoxic action of the disinfectants. BBR reduces the growth of gram-negative bacteria less efficiently than BBG at any of the tested concentrations, when mixed with Oct. 

Recently, Müller and Kramer (2008[20]) defined the biocompatibility index (BI) of antiseptic compounds. They measured the IC_50_ value of the antiseptics on fibroblast cells and divided this value with the concentration at which 99.9 % of the Gram-positive and Gram-negative bacteria are killed. A BI bigger than 1 would mean that an antiseptic compound is more toxic to the bacterial organisms in comparison with the mammalian cells. In order to quantify, in our case, the effectiveness of the different concentration combinations between BBG and Oct or BAK in antiseptic treatments, a similar BI of the two antiseptic compounds in the presence of BBG will be defined as the next step of this study.

Previously Dutot et al. (2006[5]) reported that BAK induces apoptosis in corneal and conjunctival cell lines through the activation of the P2x7 receptor. The activation of this receptor leads to pore formation and influx of Ca^2+^ and other extracellular molecules into the cell, but also to the escape of intracellular small metabolites (Chung et al., 2000[4]; Dutot et al., 2006[5]; Wang et al., 2004[29]). The exact mechanism of the protective activity of BBG and BBR is not yet known. We assume, however, that it does not involve the P2x7 receptor, because other selective P2x7 receptor antagonists, OxATP and DPPH, did not show any protection against the cytotoxic effect of these antiseptics. Recently Jo and Bean (2011[11]) showed that BBG at micromolar concentrations also causes inhibition of neuronal voltage gated sodium channels in neuroblastoma cells. The binding constants to these channels are far higher than those of the classic sodium channel blockers used in medical treatments of traumatic brain injury (Pitkanen et al., 2014[23]). Further investigations are needed to elucidate on which target BBG and BBR acts when inhibiting the toxicity of BAK or Oct on HCE cells. BBR, in contrast to BBG, was less studied in the past. This dye is often used in literature as a stain for detection of proteins in polyacrylamide gel electrophoresis. No protective effect of the dye on human cells has been described yet, nor any activity on any cellular receptors.

The chemical interaction between negatively charged dye molecules and antiseptics, which have one (BAK) or two (Oct) positive charges, could be one reason, why BBG and BBR reduces the toxicity of Oct or BAK on HCE cells. This idea is supported also by our data, where certain mixtures between the dyes and the antiseptics reduce the bacteriostatic effect of the antiseptics on Gram-negative bacteria. This interaction could result in a compound that is not anymore toxic to neither the mammalian cells nor to the Gram-negative bacteria. Interestingly, the bacteriostatic effect of the dye-preservative mixtures on Gram-positive bacteria is not affected by these interactions. In the literature it is mentioned that BBR, during the protein staining procedure, binds reversibly and especially to positively charged parts of the proteins (Tal et al., 1985[28]), with a slightly different molecular mechanism than BBG (Lee et al., 2001[14]). Casero et al. (1997[3]) described studies of the interaction of the negatively charged BBG with several types of cationic surfactants and the formation of dye-detergent premicellar aggregates at surfactant concentrations far below their critical micelle concentration. Ma et al. (2014[17]) showed that BAK is able to interact with other negatively charged dye molecules, eosin Y and eosin B and form stable dye-surfactant aggregates. Also Sütterlin et al. (2008[27]) presented studies where other negatively charged molecules, such as linear alkylbenzene sulfonate, naphthalene sulfonic acid, benzene sulfonic acid or SDS, can considerably modify the bacteriostatic effect of BAK on two Gram-negative bacterial strains, *P. putida* and *V. fischeri*. 

There are several preparations with BAK or Oct as an antiseptic already available on the market. The combinations between Oct and BBG or BBR, or BAK and BBG, proposed by us in this study, will help in marking the disinfected area and in protecting the skin from possible irritations or inflammations caused by the antiseptics. The protective effect of BBG and BBR will also allow to use higher concentrations of the antiseptics in the ready-to-use preparations. However, the daily use of BBG based solutions on skin or in eyes will have as a side effect the staining of the surface in blue, where the treatment is applied, but according to Peng et al. (2009[22]) and our own experience, the staining disappears rapidly, and is gone about a week after the treatment. With BBR no such effect has been described yet in the literature, but we assume that it has a similar staining effect with BBG, because BBR is widely used as a high sensitivity protein stain.

## Acknowledgements

The research was partially funded by the Dr. Helmut und Margarete Meyer-Schwarting Stiftung. We are also grateful for the octenidine dihydrochloride gift from Schülke and Mayr GmbH.

## Declarations

The authors declare that they have no conflict of interest.
